# Identification of Key Genes in Gastric Cancer by Bioinformatics Analysis

**DOI:** 10.1155/2020/7658230

**Published:** 2020-09-21

**Authors:** Xinyu Chong, Rui Peng, Yan Sun, Luyu Zhang, Zheng Zhang

**Affiliations:** ^1^Department of Molecular Medicine and Cancer Research Center, Chongqing Medical University, Chongqing, China; ^2^Department of Bioinformatics, Chongqing Medical University, Chongqing, China

## Abstract

Gastric cancer (GC) is one of the most common malignancies of the digestive system with few genetic markers for its early detection and prevention. In this study, differentially expressed genes (DEGs) were analyzed using GEO2R from GSE54129 and GSE13911 of the Gene Expression Omnibus (GEO). Then, gene enrichment analysis, protein-protein interaction (PPI) network construction, and topological analysis were performed on the DEGs by the Gene Ontology (GO), Kyoto Encyclopedia of Genes and Genomes (KEGG) pathway, STRING, and Cytoscape. Finally, we performed survival analysis of key genes through the Kaplan-Meier plotter. A total of 1034 DEGs were identified in GC. GO and KEGG results showed that DEGs mainly enriched in plasma membrane, cell adhesion, and PI3K-Akt signaling pathway. Subsequently, the PPI network with 44 nodes and 333 edges was constructed, and 18 candidate genes in the network were focused on by centrality analysis and module analysis. Furthermore, data showed that high expressions of fibronectin 1(FN1), the tissue inhibitor of metalloproteinases 1 (TIMP1), secreted phosphoprotein 1 (SPP1), apolipoprotein E (APOE), and versican (VCAN) were related to poor overall survivals in GC patients. In summary, this study suggests that FN1, TIMP1, SPP1, APOE, and VCAN may act as the key genes in GC.

## 1. Introduction

Gastric cancer (GC) is one of the malignant tumors threatening human health, and it is the fifth most common cancer and the third leading cause of cancer death in the world [1, 2]. The cause of the onset of it includes diet, lifestyle, genetic, and infection of helicobacter pylori[3–5]. At present, the treatment of gastric cancer includes surgery [6], radiotherapy [7], neoadjuvant chemotherapy [8], and immunotherapy [9]. The survival rate for early gastric cancer patients can reach 90%. However, it is difficult to detect and diagnose the disease during the early period, resulting in a significant decrease in survival after diagnosis [10]. Therefore, it is vitally important to explore potential diagnostic and prognostic biomarkers and therapeutic targets of early GC.

With the advance of the human genome project, cancer has been studied at the genetic level. Gene chips can be used to identify genes that cause early cancer. It has the characteristics of high flux, high sensitivity, and low cost. It is widely used in disease diagnosis and drug screening [11, 12]. At present, DNA microarrays were used to identify potential biomarkers that affect the development of diseases in studies [13]. Gene microarray technology plays an important role in elucidating cell cycle-related genes and evaluating their expression in gastric cancer [14]. However, the pathogenesis of GC remains unclear.

In this study, we aimed to obtain the key genes between GC patients and normal controls. We downloaded the gene expression profiles of GSE54129 and GSE13911 and identified 1034 differentially expressed genes (DEGs) in GC. Moreover, Gene Ontology (GO) and Kyoto Encyclopedia of Genes and Genomes (KEGG) enrichment analysis were carried out for DEGs, and GC-related protein-protein interaction (PPI) network was constructed. Furthermore, 18 candidate genes displayed high centrality values and located at the 1^st^ module, which were found by the centrality analysis and module analysis on the basis of the GC-related PPI network. Data also showed that high expressions of fibronectin 1 (FN1), the tissue inhibitor of metalloproteinases 1 (TIMP1), secreted phosphoprotein 1 (SPP1), apolipoprotein E (APOE), and versican (VCAN) were related to a poor overall survival in gastric cancer patients. These key genes could be used as potential therapeutic targets and biomarkers for gastric cancer at early period.

## 2. Materials and Methods

### 2.1. Microarray Data

The Gene Expression Omnibus database (http://www.ncbi.nlm.nih.gov/geo) is a public genome database [15]. In this study, two gene expression profiles GSE54129 and GSE13911 were downloaded from the GEO. The criteria for both gene expression profiles were (a) the samples included two groups of GC tumors and normal tissues; (b) the sample size of each gene expression profile was greater than 60; (c) they were recently updated in the last two years (2019-2020); and (d) they were derived from the same platform: GPL570 [hg-u133_plus_2] Affymetrix Human Genome U133 Plus 2.0 Array. The GSE54129 data profile contained 111 tumor tissues and 21 normal tissues. The GSE13911 data profile contained 38 tumor tissues and 31 normal tissues.

### 2.2. Identification of DEGs

GEO2R (https://www.ncbi.nlm.nih.gov/geo/geo2r/) online analysis software was used to analyze the differentially expressed genes in tumor and normal samples of GC. The intersection of DEGs was obtained by Venny 2.1.0 (https://bioinfogp.cnb.csic.es/tools/venny/) [16]. The adj. *p* value was obtained by the Benjamini and Hochberg method to control the probability of false positives. Adj. *p* value < 0.01 and ∣log FC | >1 were used as the cut-off criteria [17].

### 2.3. GO and KEGG Enrichment Analysis of DEGs

The DAVID (https://david.ncifcrf.gov/) is a database that provides systematic and comprehensive annotation information of biological functions for genes and proteins [18]. GO is an international standard classification system of gene function [19]. It includes biological process (BP), cellular component (CC), and molecular function (MF). KEGG is a database for systematic analysis of gene function and genomic information [20]. We used DAVID to carry out GO functional annotation and KEGG pathway enrichment analysis on the DEGs. *p* < 0.05 were considered statistically significant [21].

### 2.4. PPI Network Construction

The STRING (http://string-db.org) is a database for searching between known proteins and predicting the interactions between proteins [22]. We used it to build PPI network for DEGs. The combined score > 0.4 was considered as the cut-off value [23].

### 2.5. Module Analysis of PPI Network

The Cytoscape is a software for visual networks [24]. Functional modules in the network were identified by using the plug-in MCODE of Cytoscape [25]. The selection criteria were as follows: degree cut − off = 2, node score cut − off = 0.2, *k* − core = 2, and max depth = 100. Submodules were sorted by score value. The higher the score was, the stronger the protein correlation in the module was.

### 2.6. Centrality Analysis of PPI Network

Centrality analysis includes analyzing the degree, betweenness, and eigenvector of network nodes. Cytoscape plug-in CytoNCA was used to calculate the values of degree, betweenness, and eigenvector to predict the key genes [26]. Degree centrality is a measure of the importance of a single node, and it describes the number of edges connecting nodes [27]. Betweenness centrality is the shortest path through which a particular node is analyzed [28]. Eigenvector centrality takes into account the degree of itself and the degree of its neighbors [29]. The distribution characteristics of degree, betweenness, and eigenvector were determined by density analysis. The correlation between degree and betweenness, between degree and eigenvector, and between betweenness and eigenvector was calculated by taking the top 5% of the three parameters. And it was visualized by R language.

### 2.7. Survival Analysis of Hub Genes

The Kaplan-Meier plotter (KM plotter, http://kmplot.com/analysis/) is an online website survival analysis [30]. There are 21 types of cancer, including breast cancer, ovarian cancer, lung cancer, and gastric cancer. The online analysis database includes GEO, EGA, and TCGA. The KM plotter was used to reflect the survival expression of hub genes. In the KM plotter, we selected patient grouping criteria to automatically select the optimal cutoff, overall survival, and complete follow-up time for systematic and complete survival analysis. *p* < 0.01 was set as the cut-off criterion [31].

## 3. Results

### 3.1. Identification of DEGs

**Figure 1:**
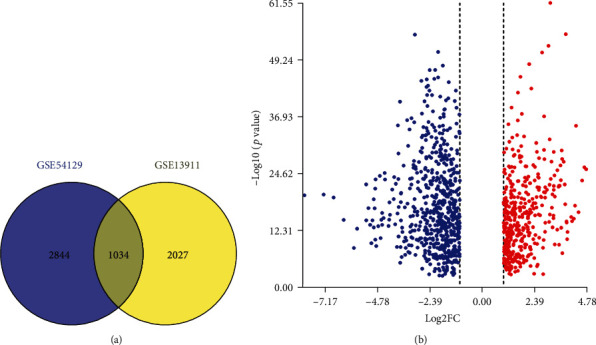
Screening and identification of differentially expressed genes. (a) Venn diagram showed the differentially expressed genes of adj. *p* value < 0.01 and ∣log2 FC | >1. (b) Red points meaned upregulated genes screened on the basis log2 FC > 1 and *p* value < 0.01. Blue points meaned downregulated genes screened on the basis log2 FC < −1 and *p* value < 0.01.

To explore the role of systems biology in the pathogenesis of GC, we analyzed two chip data of GSE54129 and GSE13911 by GEO2R. There were 3878 differentially expressed genes in GSE54129 and 3061 differentially expressed genes in GSE13911. Then, venny2.1.0 was used to obtain the intersection of the DEGs of the two chips. The results showed that 1034 differentially expressed genes appeared on both chips (Figure 1(a)). These DEGs included 403 upregulated genes and 631 downregulated genes between tumor and nontumor samples (Figure 1(b)). These data provided basic data for further analysis.

### 3.2. GO and KEGG Enrichment Analysis of DEGs

**Figure 2:**
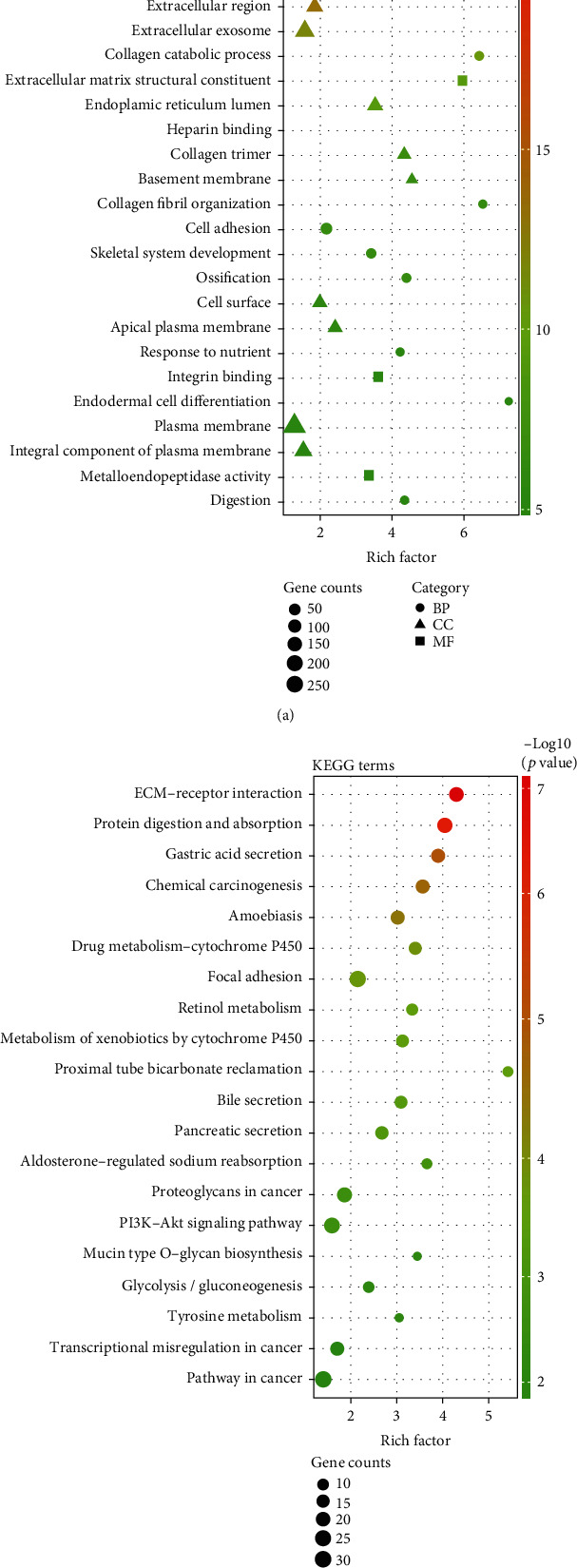
GO and KEGG enrichment analysis of the PPI network. (a) Top 25 significantly enriched gene ontology terms, including three groups (biological process, cellular component, and molecular function), *p* < 0.05. (b) The KEGG pathway analysis of DEGs in GC, *p* < 0.05.

In order to better understand the biological function of DEGs, we conducted GO function and KEGG enrichment analysis by DAVID. GO results showed that DEGs significantly enriched in extracellular matrix organization, collagen catabolic process and cell adhesion of BP, extracellular space, extracellular region and extracellular exosome of CC, extracellular matrix structural constituent, and heparin binding and integrin binding of MF (Figure 2(a)). Moreover, KEGG analysis showed that the DEGs were enriched in ECM-receptor interaction, PI3K-Akt signaling pathway and focal adhesion, and so on (Figure 2(b)).

### 3.3. Construction of GC-Related PPI Network

To study the molecular mechanism of gastric cancer from a systematic perspective, PPI network was constructed to explore the relationship between proteins. PPI network was constructed by STRING for DEGs with a confidence level of >0.4. The result of network analysis showed that PPI enrichment *p* value < 1.0*e*-16. There were 865 nodes and 4483 edges in the visualization network using the Cyctoscape.

### 3.4. Module Analysis of the PPI Network

**Figure 3:**
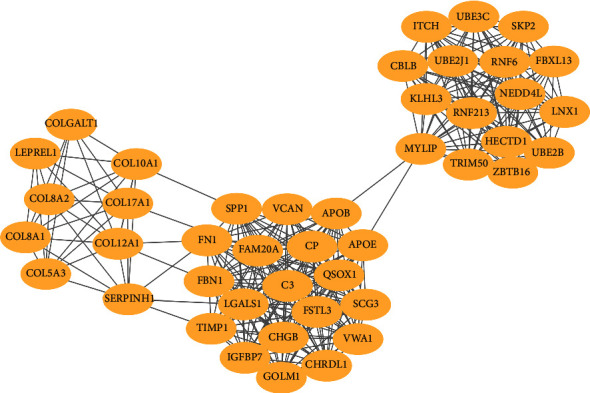
Module analysis of PPI networks obtained through Cytoscape's plug-in MCODE. The most prominent module in the PPI network included 44 nodes and 333 edges.

In order to explore more closely related genes in the complex PPI network, we conducted module analysis of the network by MCODE. The result showed that there were 27 modules in PPI network. We found that the first module was the most densely interacted region in PPI network, with a score of 15.488. The module was located at the center of the entire network, including 44 nodes and 333 edges (Figure 3). The above results suggested that the protein association in the first-rank module may be the strongest and the most significant part of the whole network.

### 3.5. Centrality Analysis of PPI Networks

**Figure 4:**
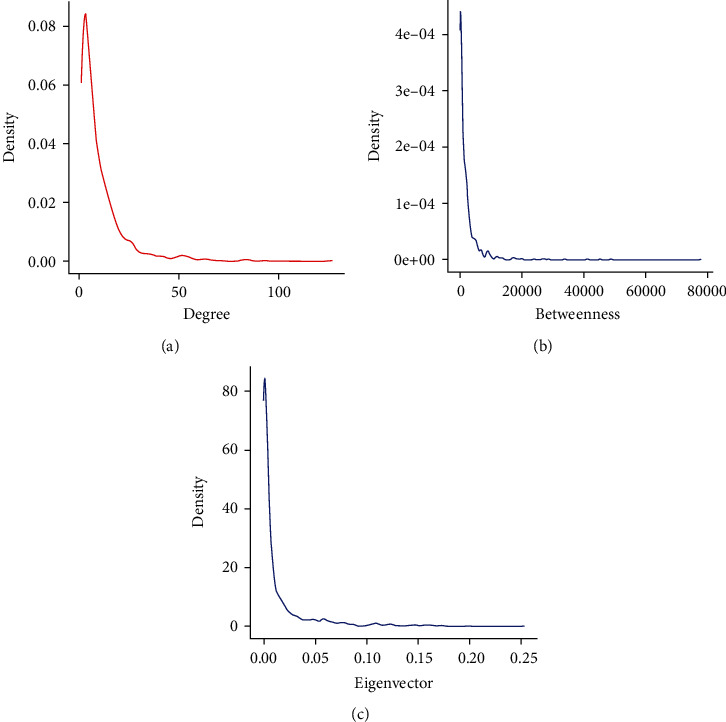
Centrality analysis of PPI networks obtained through Cytoscape's plug-in CytoNCA. (a) A density diagram of degree centrality. (b) A density diagram of betweenness centrality. (c) A density diagram of eigenvector centrality.

**Figure 5:**
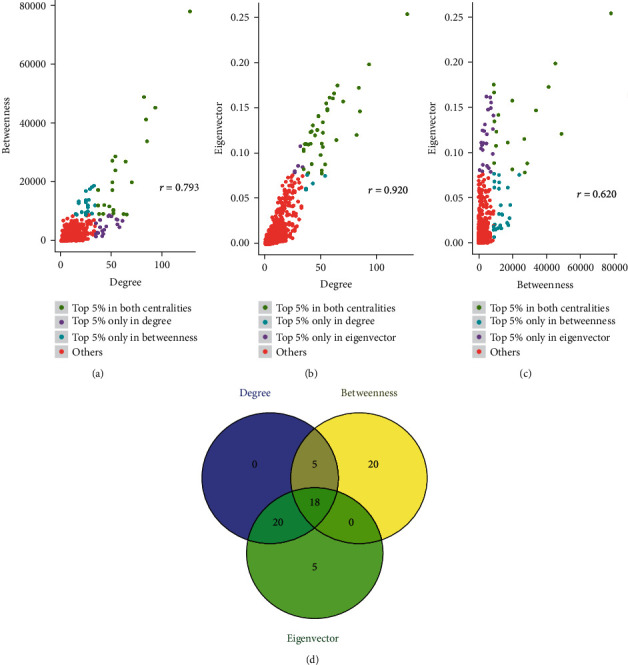
Correlation analysis of the top 5% of molecules of each centrality (degree, betweenness, and eigenvector). (a) The correlation coefficient between degree and betweenness was 0.793. (b) The correlation coefficient between degree and eigenvector was 0.920. (c) The correlation coefficient between betweenness and eigenvector was 0.620. (d) Venny2.1.0 was used to obtain the intersection of the top 5% of genes of each centrality (degree, betweenness, and eigenvector). The results showed that the 18 keys were further studied because of their high degree, betweenness, and eigenvector values.

To analyze the key genes in the complex PPI network, we used the centrality analysis to analyze them. First, we analyzed the subcases of the three parameters by their density. The results showed that degree, betweenness, and eigenvector were power-law distributions (Figure 4). Then, we took the top 5% of the three parameters and analyze their correlation. The results showed that the correlation coefficient between degree and betweenness was 0.793, the correlation coefficient between degree and eigenvector was 0.920, and the correlation coefficient between betweenness and eigenvector was 0.620 (Figures 5(a)–5(c)). The results indicated that the degree, betweenness, and eigenvector are positively correlated to each other and have significant correlations. Finally, we studied the top 5% of the genes of each parameter and obtained 18 genes with degree, betweenness, and eigenvector by taking the intersection (Figure 5(d), Table 1). Combined these with the results of module analysis, FN1, TIMP1, SPP1, apolipoprotein B (APOB), APOE, VCAN, and complement C3 (C3) were focused on because these seven genes with high centrality values were located in the first-rank module.

### 3.6. Survival Analysis of Hub Genes

**Figure 6:**
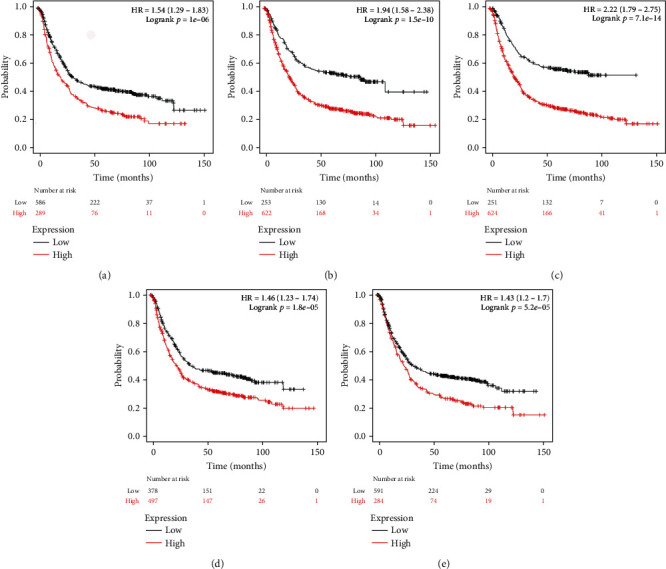
Survival analysis of key genes by the KM plotter in gastric cancer. (a) Gastric cancer patients with high expression of FN1 had poor prognosis. (b) Patients of gastric cancer with high expression of TIMP1 had poor prognosis. (c) Patients of gastric cancer with high expression of SPP1 had poor prognosis. (d) Patients of gastric cancer with high expression of APOE had poor prognosis. (e) Patients of gastric cancer with high expression of VCAN had poor prognosis (*p* < 0.01).

Survival analysis of seven candidate genes was further studied using the KM plotter. The results showed that FN1, TIMP1, SPP1, APOE, and VCAN were related to OS in gastric cancer patients (*p* < 0.01) (Figure 6).

## 4. Discussion

Microarray technology is a product of the gradual implementation of the human genome project and the rapid development and application of molecular biology. With the rapid development of gene microarray technology, people can quickly measure the expression levels of thousands of genes simultaneously [32, 33]. DNA microarray has a high-throughput speed, the characteristics of high sensitivity, and so on. It is widely used to study gene expression in a variety of organisms, including yeast, plants, and humans [34]. DNA microarrays can effectively identify disease-related genes and use them as biomarkers for diagnosis and treatment [35], including hepatocellular cancer [36], renal cell cancer [37], and colorectal cancer [38]. It has been reported that the methylation status of CpG island can be detected by microarray method, which is helpful for cancer diagnosis and detection of recurrence [39]. And studies have shown that through the microarray analysis between test specimens of gastric cancer and adjacent nontumor specimens, circRNA expression changes reveal the circRNA potential role in gastric cancer [40]. The above studies suggest that we can provide useful information for elucidating the development mechanism of gastric cancer and searching for new therapeutic targets and biomarkers through microarray technology.

In this study, we screened a total of 1034 differentially expressed genes from GSE54129 and GSE13911 gene expression profiles, among which 403 genes were upregulated, and 631 genes were downregulated. The KEGG pathway enrichment analysis revealed that the DEGs were mainly in the pathways in cancer and PI3K-Akt signaling pathway. Previous studies had shown that gastric cancer cell proliferation can be promoted by activating PI3K/Akt signaling pathway [41].

To explore the pathogenesis of gastric cancer, we constructed PPI network for systematic analysis. In the STRING database, we set the minimum interaction score with parameters >0.400 to obtain the PPI network of protein interactions. This setup avoided noise and incomplete data affecting the PPI network. MCODE discovers dense regions in PPI networks based on connection data. This function is not affected by the false-positive effect of high-throughput technology. We selected the parameters degree cut − off = 2, node score cut − off = 0.2, *k* − core = 2, and max depth = 100 to find out all the modules that interact in the network, so as to further explore the most powerful proteins in the network. Through the module analysis of the network, we found that there were 27 modules in the network. The first-rank module with a score of 15.488 was the most closely related module in the whole network. Previous studies had shown that the related genes can be screened out more accurately by modular analysis, including cervical cancer [42], glioblastoma multiforme [43], and squamous cell cancer of head and neck [44]. These indicated that modular analysis played an important role in screening molecular markers. The genes in the module with higher score were the key genes that affected the occurrence of disease.

In order to further analyze the whole PPI network, the centrality analysis was used to explain the importance of the nodes in the network and the influence of the nodes on the network. We obtained 18 genes with high central values. Moreover, seven of the 18 genes were located in first-order module. Previous studies had shown that central nodes connected more protein-protein interactions, and central nodes also had more information for path enrichment analysis, which had a notable effect in the whole network [45, 46]. The results suggested that these genes may play significant roles in gastric cancer.

The study of cancer survival analysis plays an important role in the evaluation of cancer prevention measures [47]. Our results showed that the abnormal expression of FN1, TIMP1, SPP1, APOE, and VCAN influenced the prognosis of patients with gastric cancer. The high expression of them was associated with the worse OS in gastric cancer. FN1 is a member of the glycoprotein family and is widely expressed in many cell types. It played an important role in cell adhesion, growth, migration, and differentiation [48]. Studies had shown that inhibition of the expression of FN1 can inhibit the invasion and migration of gastric cancer cells [49, 50]. TIMP1 belongs to the TIMP gene family. This gene family encodes proteins that are natural inhibitors of matrix metalloproteinases (MMPS) [51]. TIMP1 can promote the aggregation of tumor-associated fibroblasts in the body and promote the proliferation and migration of cancer cells, and has antiapoptotic function [52, 53]. Studies had shown that TIMP1 was overexpressed and promoted cell proliferation in patients with gastric cancer through the NF-*κ*B-dependent mechanism [54]. SPP1, also known as osteopontin (OPN), is an acidic glycoprotein that secretes several functions. OPN participated in the epithelial mesenchymal metastasis (EMT) pathway and played an important role in tumor metastasis [55]. Previous studies had shown that the high expression of OPN was closely related to the occurrence of gastric cancer [56]. APOE acts as the member of the family of low density lipoprotein (LDL) receptor ligands and interacts with the cell membrane receptor, which involves in cholesterol and other lipid transport [57]. APOE activated the PI3K/AKT/mTOR signaling pathway and played an important regulatory role in angiogenesis, tumor cell growth, and metastasis [58, 59]. A study had shown that tumor-associated macrophages (TAMs) interacted with gastric cancer cells through APOE to promote the migration of gastric cancer cells [60]. However, there are few reports of VCAN in gastric cancer. VCAN is a chondroitin sulfate proteoglycan. The interaction between extracellular matrix and cell surface proteins promoted cell growth, proliferation, and differentiation [61]. A study had shown that upregulation of VCAN promoted the migration and invasion of ovarian cancer cells by activating the NF-*κ*B signaling pathway [62]. The growth of renal cell cancer cells can be inhibited by the activation of TNF signaling pathway through the silencing gene VCAN [63]. In addition, VCAN stimulated mesothelioma growth by weakening the antitumor activity of macrophages [64]. The above conclusions indicated that in GC, VCAN may affect the disease progression of patients through these paths, which were worth further study. The above results indicated that these five genes were closely related to the prognosis of patients with gastric cancer and can be used as a biomarker for GC.

## 5. Conclusion

In this study, 1034 differentially expressed genes were identified. On based of these genes, GO and KEGG results showed they were mainly in plasma membrane, cell adhesion, and PI3K-Akt signaling pathway. Moreover, 18 topological key genes of the 1^st^-rank module were focused on. Furthermore, five of them (FN1, TIMP1, SPP1, APOE, and VCAN) were found to be related to gastric cancer. Therefore, it provides new research directions for the detection and treatment of gastric cancer. However, their involvement in the molecular mechanisms of disease needs further clinical studies.

## Figures and Tables

**Table 1:** Top 5% of candidate genes in the centrality analysis.

Gene	Degree	Betweenness	Eigenvector
FN1	127	77922.220	0.253395320
MMP9	93	45189.664	0.198073490
CXCL8	85	33816.297	0.146214620
CD44	84	41183.110	0.172110660
MYC	82	48818.060	0.120246940
CXCL12	70	19844.107	0.157128860
TIMP1	65	8912.090	0.174682420
PTGS2	64	26884.875	0.114611500
SPP1	62	9094.989	0.165916760
APOB	54	28650.291	0.087770930
VCAN	52	9312.416	0.134104030
ICAM1	52	10434.576	0.122722970
CXCL1	52	10233.371	0.106985554
APOE	51	17188.280	0.110692450
STAT1	51	19835.434	0.081156254
KRAS	51	27196.115	0.077731330
BGN	48	11646.911	0.141241250
C3	42	9121.711	0.088115714

## Data Availability

The GSE54129 and GSE13911 data used to support the findings of this study are included within the article.
